# Characterization of Three Different Endolysins Effective against Gram-Negative Bacteria

**DOI:** 10.3390/v15030679

**Published:** 2023-03-04

**Authors:** Tae-Hwan Jeong, Hye-Won Hong, Min Soo Kim, Miryoung Song, Heejoon Myung

**Affiliations:** 1 Department of Bioscience and Biotechnology, Hankuk University of Foreign Studies, Yong-In 17035, Gyung-Gi Do, Republic of Korea; 2 LyseNTech Co. Ltd., Seongnam 13486, Gyung-Gi Do, Republic of Korea; 3 The Bacteriophage Bank of Korea, Seongnam 13486, Gyung-Gi Do, Republic of Korea

**Keywords:** bacteriophage, endolysin, Gram negative, in vivo efficacy, intrinsic efficacy

## Abstract

Genes encoding endolysins were identified and cloned from three different *Escherichia coli* bacteriophages, 10-24(13), PBEC30, and PBEC56. Putative antimicrobial peptide (AMP)-like C-terminal alpha helix structures with amphipathic natures were predicted from the three endolysins. Each gene was cloned and expressed as hexahistidine-tagged forms, and the products were purified and characterized. The purified endolysins exhibited antibacterial activities against a variety of Gram-negative bacteria including *Escherichia coli*, *Pseudomonas aeruginosa*, *Acinetobacter baumannii*, and *Klebsiella pneumonia*. Their antibacterial activities were improved by N-terminal fusion with an antimicrobial peptide, cecropin A. Minimum inhibitory concentrations (MIC) were as low as 4 μg/mL, depending on the targeted strain. The endolysins’ enzymatic activities were not affected by changes in pH at ranges from 5 to 10 and were stable at temperatures between 4 and 65 °C. The in vivo efficacies of the three endolysins were also demonstrated using *Galleria melonella* for infection models.

## 1. Introduction

The rapid proliferation of antibiotic resistant pathogens represents a global health threat [1,2,3]. In particular, ESKAPE pathogens (*Enterococcus faecium*, *Staphylococcus aureus*, *Klebsiella pneumoniae*, *Acinetobacter baumannii*, *Pseudomonas aeruginosa*, and *Enterobacter* species) pose a significant danger [4]. Despite this, the development of novel antibiotics has been extremely slow [5,6].

Endolysins are bacteriophage-encoded lytic enzymes [7,8,9]. Assembled phage particles burst out through a holin-induced passage formed between the inner membrane and the degraded space in the cell wall [10]. Recombinant endolysins can externally attack bacteria, degrading the cell wall and thus acting as antimicrobials. Many endolysins targeting Gram-positive pathogens including *Staphylococcus aureus* [11,12,13,14], *Enterococcus faecalis* [15], and *Bacillus cereus* [16] have been isolated and characterized.

Unlike Gram-positive bacteria, where the peptidoglycan cell wall is the outermost structure, Gram-negative bacteria are surrounded by an outer membrane. Large molecules such as proteins cannot pass through the outer membrane, which is a protective barrier of bacteria. This hampers most recombinant endolysins, preventing the effective induction of cell lysis by blocking entry through the membrane and subsequent contact with the cell wall. However, a few exceptional cases have been reported where the intrinsic antibacterial activity of native endolysins was demonstrated in vitro and/or in vivo. Of the latter, these include LysPA26 against *P. aeruginosa* [17], Ply6A3 against *A. baumannii* [18], Ts2631 from *Thermus scotductus* phage vB_tsc2631 [19], LysSS against various gram-negative pathogens [20], LysAB54 against a number of Gram-negative pathogens [21], engineered Artilysin [22,23], engineered endolysin LysMK34 [24], *Myoviridae* Bacteriophage Lysins LysECD7 and LysAm24 [25], ST01 against Gram negative pathogens [26], cell-penetrating peptide-fused PA90 [27], and engineered endolysin LNT113 [28]. Interestingly, endolysins exhibiting intrinsic antibacterial efficacy harbor an antimicrobial peptide (AMP)-like amphipathic alpha-helical structure at the C-terminus [29,30]. It is thought that the AMP-like helix assists the entry of the whole protein through the outer membrane. These endolysins also demonstrate a broad range of activity against targeted Gram-negative bacteria.

This study reports on the isolation and characterization of three novel endolysins with intrinsic antibacterial activity against a variety of Gram-negative bacteria.

## 2. Materials and Methods

### 2.1. Bacterial Strains and Growth Media

*Escherichia coli*, *Acinetobacter baumannii*, and *Enterobacter cloacae* strains with ATCC numbers were obtained from the American Type Culture Collection (ATCC). *Acinetobacter baumannii* and *Klebsiella pneumoniae* strains with KCTC numbers were obtained from the Korean Collection for Type Cultures (KCTC). Strains with names starting with F were isolated from clinical patients and were kind gifts from Professor Kwan Soo Ko (Sungkyunkwan University). Strains with names commencing with K were isolated from clinical patients and generously donated by Professor Min Sang Shin (Kyungbuk National University). For cloning purposes, *Escherichia coli* DH5a was used. For the overexpression of proteins, *E. coli* strains BL21(DE3) pLysS (Invitrogen, Seoul, Republic of Korea), or BL21(DE3) Star (Invitrogen, USA) were used. Bacteria were grown in either Luria Bertani (LB) medium (Duchefa Biochemie, Haarlem, The Netherlands) or CAA medium (5 g/L Casamino acids, 5.2 mM K_2_HPO_4_, and 1 mM MgSO_4_).

### 2.2. Bacteriophage Cultures

Phages 10-24(13), PBEC30, and PBEC56 were obtained from the Bacteriophage Bank of Korea (www.phagebank.or.kr, accessed on 15 January 2020). The host bacterium used was *E. coli* (ATCC 8739). Phages were mixed with freshly grown exponential phase bacteria (with 0.4 OD^600^) at an MOI of 0.001 and the mixture was incubated at room temperature for one hour for phage adsorption. The mixture was moved to a 37 °C shaking incubator and further incubated for three hours. After incubation, chloroform was added to the mixture at a final concentration of 5% (volume/volume) for complete lysis of the remaining bacteria. Then, NaCl was added to the mixture at a final concentration of 6% (weight/volume) and the mixture was incubated at 4 °C for one hour. To remove any bacterial debris, the mixture was subjected to centrifugation at 11,000× *g* for ten minutes. The supernatant was collected and polyethylene glycol 8000 was added at a final concentration of 10% (weight/volume). After centrifugation at 11,000× *g* for ten minutes, the supernatant was discarded and the pellet was resuspended in 1 mL SM buffer (50 mM Tris-HCl, pH 7.5, 100 mM NaCl, 8 mM MgSO_4_).

### 2.3. Phage DNA Isolation and In Silico Analysis

A Phage DNA Isolation kit (Norgen Thorold, ON, Canada) was used to isolate phage genomic DNA. Whole genome sequencing was performed using Illumina Miseq (LAS, Seoul, Korea). Genome assembly was performed using a SEVAGE. ORF Finder, generating three putative endolysins; Lys10-24(13) (GenBank accession no. OM650690), LysPBEC30 (GenBank accession no. OM650691), and LysPBEC56 (GenBank accession no. OM650692). Structural prediction of proteins was performed using Iterative Threading ASSEmbly Refinement (I-TASSER, https://zhanggroup.org/I-TASSER/, accessed on 1 October 2022). Predicted 3D structures were generated using 3D viewer Mol^*^ (Protein Data Base, https://www.rcsb.org/docs/3d-viewers/mol*/getting-started, accessed on 8 october 2022). As a negative control, an endolysin from a phage infecting *E. coli*, LysEC508M, which lacked the C-terminal amphipathic helix (Supplementary Figure S1) was used.

### 2.4. Cloning of Three Endolysin Genes for Expression

The genes were cloned natively or in N-terminal cecropin-fused forms. The primers used for Lys10-24(13) were forward, gcgcGGATCCATGAATATATTTGAAATGTTACGT, and reverse, gcgcCTCGAGTAGATTTTTATACGCGTCCCAAGT; for LysPBEC30 they were forward, gcgcGGATCCATGCGATTCAGTGATA, and reverse, gcgcCTCGAGCGCCGCGTTACG; and for LysPBEC56 they were forward, gcgcGGATCCATGCAACTCTCAAGAAAA, and reverse, gcgcCTCGAGCTTTGGATATACACTGTCAAGATAA ATGTCAG. When necessary, the coding sequence for cecropin A (NCBI PRF 0708214A) was fused at the terminus of each endolysin with a flexible linker (GSGSGS × 3). The resulting PCR product was cloned into the vector pET21a+ (Novagen, Thorold, ON, Canada) using the BamHI (Promega, Seoul, Republic of Korea) and XhoI (Promega, Seoul, Republic of Korea) sites for the expression of the hexahistidine-tagged protein.

### 2.5. Overexpression and Purification of Endolysins

Native LysPBEC56 was purified from *E. coli* BL21 (DE3) pLysS. All other endolysins were purified from *E. coli* BL21 (DE3) Star. The bacteria were cultured in 1 L of LB broth at 37 °C in the presence of ampicillin and/or chloramphenicol until reaching the exponential phase (0.5 OD^600^). Isopropyl-β-D-thiogalactopyranoside (IPTG) was added to the culture at a final concentration of 1 mM and the culture was further incubated for three hours for induction. Then, cells were harvested by centrifugation at 5000× *g* for 10 min. The pellets were recovered and resuspended in 50 mL lysis buffer (20 mM Tris-HCl, pH 7.5, 500 mM NaCl, and 20 mM imidazole). The mixture was then subjected to sonication to disrupt the cells and filtered through 0.45 μM pore sized filters (GVS, Zola Predosa, Italy). The filtrate was loaded on a HisTrap HP column (Cytiva), and the protein was affinity-purified using the ÄKTA Go Fast protein liquid chromatography (FPLC) system (Cytiva). Affinity-trapped endolysin was eluted with elution buffer (20 mM Tris-HCl, pH 7.5, 0.5M NaCl) containing imidazole at concentrations of between 15 and 500 mM in a gradient. Eluted fractions were subjected to cation exchange chromatography on a HiTrap SP column (Cytiva). Proteins were bound to the resin with Tris buffer (20 mM Tris-Hcl; pH 7.5) and elution was performed with Tris buffer containing NaCl (20 mM Tris-Hcl; pH 7.5, 1M NaCl). The obtained protein was dialyzed in 1X phosphate buffered saline (PBS) using a Pur-A-Lyzer^TM^ Mega 6000 Dialysis Kit (Sigma, Seoul, Republic of Korea) at 4 °C overnight. Purified proteins were quantitated using a Bradford assay kit (Bio-Rad, Seoul, Republic of Korea).

### 2.6. Zymogram Assay

An amount of 100 mL overnight-cultured *E. coli* ATCC8739 was harvested and washed once with PBS, followed by centrifugation at 4000× *g* for 15 min. The recovered pellet was resuspended in 3 mL deionized water and autoclaved. The resulting solution was used to make 10 mL of 15% SDS polyacrylamide gel. Then, 5 μg of purified endolysin was loaded onto SDS-PAGE and electrophoresis performed. The gel was then washed in deionized water for one hour followed by incubation in the reaction buffer (1% Triton X-100, 20 mM Tris–HCl, pH 7.5) at 37 °C until clear zones appeared.

### 2.7. Colony Forming Unit (CFU) Reduction Assay

Each endolysin’s ability to kill targeted bacteria was measured by counting the number of viable bacteria after incubation with endolysin. Each target bacterium was freshly cultured to the exponential phase (OD^600^ 0.5) and harvested with centrifugation at 11,000× *g* for one minute. The pellet was washed and resuspended in reaction buffer (20 mM Tris-Hcl; pH 7.5) and the cells were diluted to 10^7^/mL. Then, 100 mL of cell suspension and each endolysin was mixed to a final concentration of 0, 0.125, 0.25, or 0.5 μM. The mixture was incubated at 37 °C for two hours, followed by viable cell counting on LB agar plates.

### 2.8. Stability Test of Endolysins

For pH stability, each endolysin was preincubated in 20 mM Tris-Cl buffer, pH 5.0, 6.5, 7.5, 8.5, or 10 at room temperature for one hour before testing. For temperature stability, each endolysin was also preincubated in 20 mM Tris-Cl buffer, pH 7.5 at 4, 25, 37, 45, 55, 65, or 80 °C for one hour before testing. Preincubated endolysins were subjected to CFU reduction assay, as described above.

### 2.9. Determination of Minimum Inhibitory Concentration (MIC)

Broth microdilution in 96 well plates, according to a previously described method [26], was performed. Briefly, overnight cultured bacteria were transferred to CAA medium (5 g/L casamino acids, 5.2 mM K_2_HPO_4_, and 1 mM MgSO_4_) and freshly incubated at 37 °C for three hours. Cells were dispensed at 1 × 10^4^ cells/well containing 100 mL of CAA medium in a 96 well plate and purified endolysin was added to each well at concentrations of 1 to 64 μg/mL with 1/2 serial dilutions. The mixture was incubated at 37 °C for 20 h. The minimum concentration of endolysin in a well which resulted in the complete inhibition of bacterial growth was determined to be the MIC.

### 2.10. Galleria Mellonella Infection Model

Final instar stage larvae of *Galleria mellonella* were obtained from Sworm (Chonan, Korea). Before bacterial infection, the larvae were incubated without food at 30 °C for 24 h. *A. baumannii* ATCC19606 were fresh cultured for three hours and subjected to a centrifugation at 4000× *g* for three minutes. The pellet was recovered and resuspended in PBS. Then, 5 mL of 5 × 10^6^ CFU of bacteria and 5 mL of 5 μg/mL endolysin were mixed and 10 mL of the mixture was injected into the last-left-proleg of each larva using a 10 RGT 10 mL syringe (Trojan Scientific and Medical, Ringwood, Victoria, Australia). The larvae were incubated at 30 °C for 96 h. Four animal groups consisting of ten larvae were used; group 1, positive control group with 10 mL PBS treatment; group 2, negative control group with 5 mL PBS + 5 mL of 5 × 10^6^ CFU/mL bacteria; group 3, 5 mL of 5 μg/mL native endolysin + 5 mL of 5 × 10^6^ CFU/mL bacteria; and group 4, 5 mL of 5 μg/mL cecA-fused endolysin + 5 mL of 5 × 10^6^ CFU/mL bacteria.

### 2.11. Statistical Analysis

Data analysis was performed using GraphPad Prism (version 9.3.0). In vitro experiments were carried out in triplicate and the two-tailed Student *t*-test was used for statistical analysis. In vivo experiments were analyzed using the log-rank (Mantel-Cox) test.

## 3. Results

**Figure 1:**
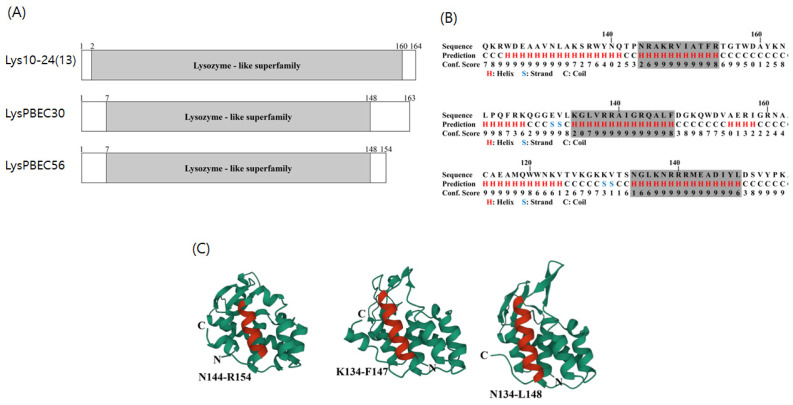
Amino-acid sequence-based structure prediction of the three endolysins. (**A**) Predicted lysozyme-like superfamily domains are shown for each endolysin. (**B**) Predicted secondary structure of C-terminal region of each endolysin using I-Tasser. Shaded sequences denote an alpha helix with an amphipathic nature. (**C**) Predicted 3D structure generated using 3D viewer Mol^*^. Alpha helix with an amphipathic nature is shown in red, with amino acid positions given in numbers.

The predicted domain structures of the three endolysins are illustrated in Figure 1A. Each harbored an enzymatically active domain (EAD) characteristic of the lysozyme-like superfamily. No cell wall binding domain (CBD) was found. The predicted amphipathic helix with hydrophobic amino acids and cationic amino acids at opposite sites was mapped at amino acids 144–154 of Lys10-24(13), 134–147 of LysPBEC30, and 134–148 of LysPBEC56 (Figure 1B). The predicted three dimensional structures are shown in Figure 1C. The three endolysins appeared to be members of the group harboring an antimicrobial peptide (AMP)-like amphipathic helix at the C-terminus, demonstrating intrinsic antibacterial activity against Gram-negative bacteria [29,30].

**Figure 2:**
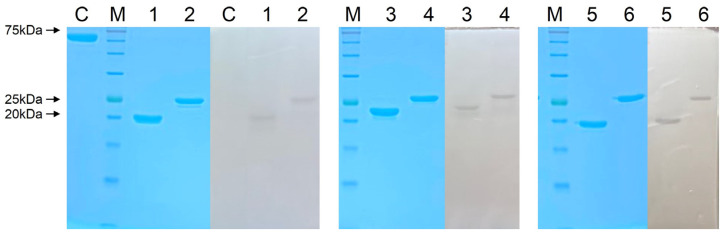
Purification and zymogram analysis of each endolysin. Three endolysins were purified to near homogeneity (shown in color) and a zymogram assay performed (shown in black and white). C, Bovine serum albumin (BSA) used as a control; M, molecular weight marker; 1, LysP10-24(13); 2, CecA-LysP10-24(13); 3, LysPBEC30; 4, CecA-LysPBEC30; 5, LysPBEC56; 6, CecA-LysPBEC56.

The native endolysins, or those fused to cecropin A (an antimicrobial peptide) at their N-termini, were purified in hexahistidine-tagged forms to near homogeneity using Ni-NTA affinity chromatography followed by cation exchange chromatography (Figure 2). N-terminal fusion of cecropin A [31,32] has been seen to greatly improve the antibacterial activity of native endolysins in previous studies [24,28]. Thus, two different versions of each endolysin were constructed and purified: a native form and an N-terminal cecropin A-fused form. The cell wall-degrading activities were initially observed by performing a zymogram assay on the purified endolysins (Figure 2).

**Figure 3:**
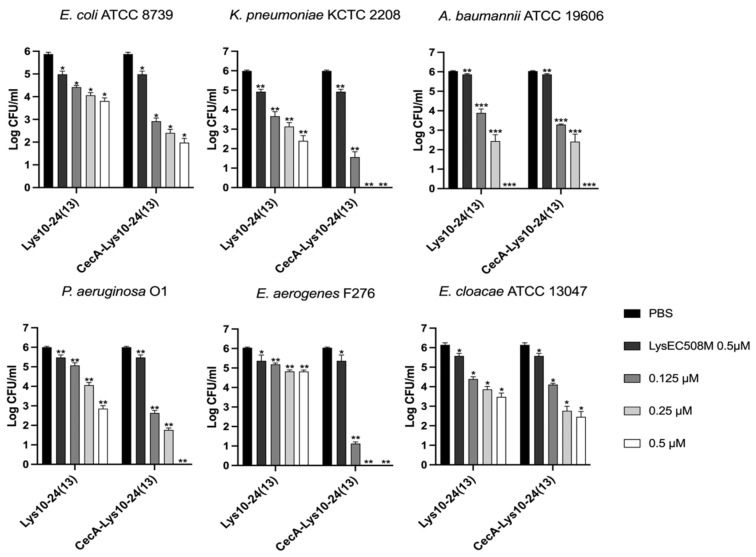
Antibacterial activity of Lys10-24(13) against various Gram-negative pathogens. Six different ESKAPE pathogens were tested. Lys10-24(13) or CecA-Lys10-24(13) were added to the final concentrations indicated and the mixture were incubated for two hours before viable colony counting. LysEC508M which lacked the C-terminal amphipathic helix, without fusion to cecropin A, was used as a negative control. Experiments were performed in triplicate. *** *p* < 0.001, ** *p* < 0.01, * *p* < 0.05.

**Figure 4:**
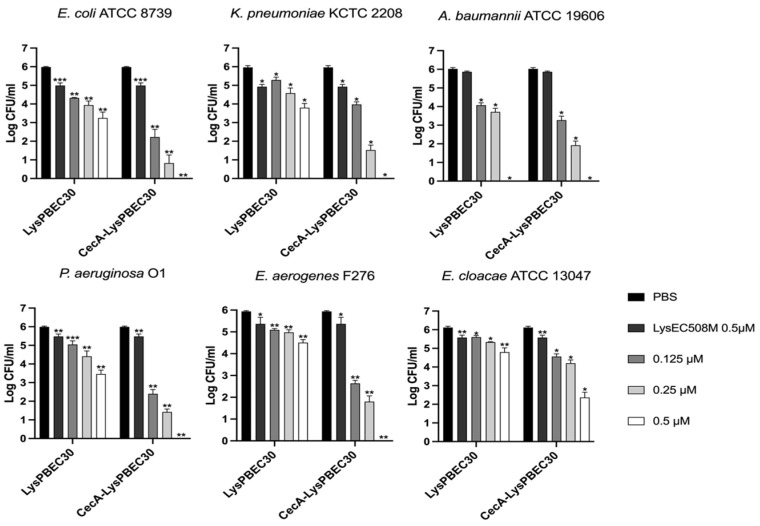
Antibacterial activity of LysPBEC30 against various Gram-negative pathogens. Six different ESKAPE pathogens were tested. LysPBEC30 or CecA-LysPBEC30 were added to the final concentrations indicated and the mixtures were incubated for two hours before viable colony counting. LysEC508M which lacked the C-terminal amphipathic helix, without fusion to cecropin A, was used as a negative control. Experiments were performed in triplicate. *** *p* < 0.001, ** *p* < 0.01, * *p* < 0.05.

**Figure 5:**
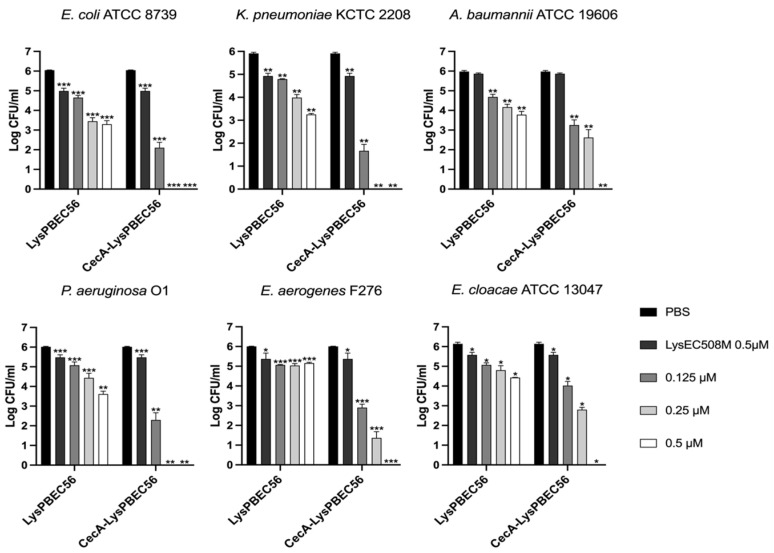
Antibacterial activity of LysPBEC56 against various Gram-negative pathogens. Six different ESKAPE pathogens were tested. LysPBEC56 or CecA-LysPBEC56 were added to the final concentrations indicated and the mixtures were incubated for two hours before viable colony counting. LysEC508M which lacked the C-terminal amphipathic helix, without fusion to cecropin A, was used as a negative control. Experiments were performed in triplicate. *** *p* < 0.001, ** *p* < 0.01, * *p* < 0.05.

The antibacterial efficacy of the purified endolysins was observed against *E. coli, P. aeruginosa*, *A. baumannii*, *K. pneumonia*, *E. cloacae*, and *E. aerogenes* in vitro (Figure 3, Figure 4 and Figure 5). Overall, the presence of cecropin A at the N-terminus of each endolysin improved antibacterial efficacy significantly. Also, the endolysins killed target bacteria in a dose-dependent manner. CecA-Lys10-24(13) was the most effective against *E. aerogenes*, followed by *K. pneumoniae* (Figure 3). The presence of cecropin A at the N-terminus conferred the greatest advantage when targeting *E. aerogenes*. The effect was least observed when targeting *A. baumannii* and *E. cloacae*. CecA-LysPBEC30 was most effective against *E. coli* (Figure 4). The presence of cecropin A at the N-terminus conferred the greatest advantage when targeting *E. aerogenes*. The effect was least observed when targeting *A. baumannii*. CecA-LysPBEC56 was most effective against *E. coli and K. pneumoniae* (Figure 5). The presence of cecropin A at the N-terminus conferred the greatest advantage when targeting *E. aerogenes*. The effect was least observed when targeting *A. baumannii* or *E. cloacae*. Interestingly, all three endolysins benefitted the most from cecropin A fusion when targeting *E. aerogenes*. Also, all three endolysins benefitted the least from cecropin A fusion when targeting *A. baumannii*.

The minimum inhibitory concentration (MIC) is the minimum concentration of drug in which target bacteria do not grow in a serial 1/2 dilution plate. We checked the MICs for each endolysin against various strains of Gram-negative bacteria (Table 1). The MIC was as low as 4 μg/mL for each endolysin. Based on the number of test strains with MICs > 64 μg/mL, CecA-Lys10-24(13) was the most effective, followed by CecA-PBEC56 and CecA-PBEC30. Overall, 30.6% of *P. aeruginosa* strains, 30.3% of *E. coli* strains, 25.0% of *K. pneumoniae* strains and only 2.6% of *A. baumannii* strains tested had MICs > 64 mg/mL.

**Figure 6:**
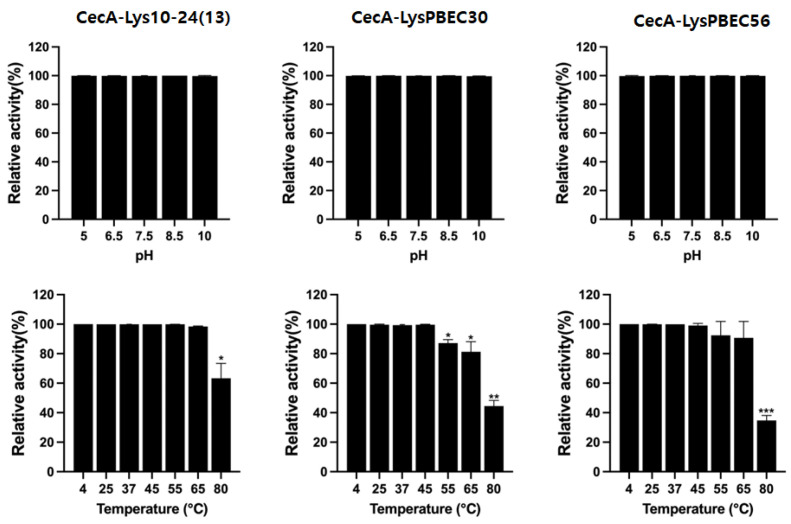
Stability of each endolysin at indicated pH or temperature. Residual activity of endolysins after exposure at indicated pH or temperature for one hour was measured by CFU reduction assay. Experiments were performed in triplicate. *** *p* < 0.001, ** *p* < 0.01, * *p* < 0.05.

All three endolysins retained their activity after an hour of incubation at pHs ranging from between 5 and 10 (Figure 6), but their thermal stability varied. CecA-Lys10-24(13) and CecA-LysPBEC56 were stable after an hour of exposure to 65 °C, while CecA-LysPBEC30’s stability began to decline after exposure to 55 °C.

**Figure 7:**
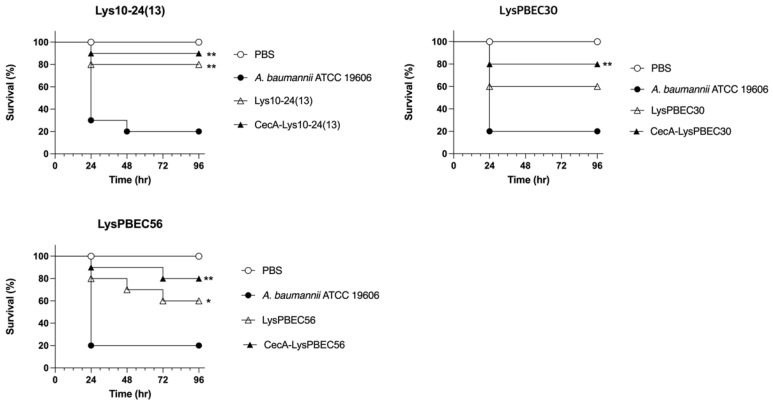
In vivo efficacy of each endolysin using *Galleria mellonella* infection model. * *p* < 0.05, ** *p* < 0.01.

In vivo efficacy tests were performed for the three endolysins using *Galleria mellonella* for animal modeling (Figure 7). The larvae were infected with *A. baumannii* and treated with each endolysin, followed by observation of survival for 96 h. Most of the untreated larvae died 24 h post infection. Treatments with cecropin A-fused endolysins increased the survival significantly, by ≥4 fold. The efficacy of the wild type endolysins was also proven, but not to the extent of the cecropin A-fused forms. It is notable that the in vivo efficacy of Lys10-24(13) was better than that of the others, consistent with MIC (Table 1) and temperature stability (Figure 6) results.

The animals were divided into four groups. PBS, those injected with PBS without infection; *A. baumanni* ATCC19606, those infected with the bacteria; Lys10-24(13), those infected with the bacteria and treated with the wild type endolysin; CecA-Lys10-24(13), those infected with the bacteria and treated with CecA-fused endolysin. The same for treatment with LysPBEC30 and LysPBEC56. Each group contained 10 larvae. ** *p* < 0.01, * *p* < 0.05.

## 4. Discussion

There have been previous reports aimed at the study of a single endolysin targeting Gram-negative pathogens. However, a comparison of unrelated endolysins targeting bacteria, in particular one incorporating the inclusion of in vivo efficacy data, has never been previously reported due to the scarcity of such endolysins. In this study, we were able to compare a variety of aspects of three different endolysins including the presence or absence of a C-terminal alpha helix with an amphipathic nature, stabilities, and efficacies both in vitro and in vivo.

We confirmed the presence of a C-terminal alpha helix with an amphipathic nature, further supporting the hypothesis that this is a common feature in endolysins harboring intrinsic antibacterial activity when supplied as recombinant proteins, consistent with the findings of previous studies [29,30].

Lys10-24(13) was effective against most of the bacteria and bacterial strains tested, except for one or two specific strains of *E. coli*, *P. aeruginosa*, and *A. baumannii*. LysPBEC30 was most effective against *A. baumannii*, but not as effective against strains of other bacteria. LysPBEC56 exhibited similar characteristics, except that it was more effective against *K. pneumoniae* than LysPBEC30. Accordingly, we can conclude that the efficacy of each endolysin depends on the specifically targeted bacteria and strains. Nevertherless, we could find some common features of the endolysins in terms of efficacy. Lys10-24(13) and LysPBEC30 possesed higher antibacterial activities against *A. baumannii* than other pathogens in their native form without fusion of cecropin A. It is thought that the cationic part of the C-terminal amphipathic helix interacts with anionic phosphate groups of lipd A present in the outer membrane, facilitating initial contact of the endolysin to target bacteria. Then the hydrophobic part of the amphipathic helix may induce the entrance of the endolysin through the membrane [33,34]. The outstanding efficacies of the two native endolysins may be explained based on the outer membrane structure of *A. baumannii* compared to other Gram negatives. The area per lipid (APL) of phosphorylated lipd A in *A. baumanni* was wider than that of P. aeruginosa or E. coli, and comaparable to that of K. pneumoniae, while the membrane thickness (Tmemb) of *A. baumannii* was thicker than *P. aeruginosa*, but thinner than *E. coli* or *K. pneumoniae* [35]. Thus, stronger initial attachment of the endolysins to lipid A and the following passage through thinner membrane may be the reason why we could observe better antibacterial efficacies of the two endolysins agaist *A. baumannii*.

Another common feature is that the fusion of cecropin A enhanced antibacterial activities of all three endolysins against all target pathogens tested. Cecropin A itself possesses a cationic part which faciliatates interaction with lipid A. Thus, a stronger ionic interaction is anticipated in the presence of the antimicrobial peptide for the endolysins. Once the fusion endolysin degrades the cell wall, the antimicrobial peptide may damage the inner membrane, further enhancing the antimicrobial efficacy.

Our observation that the temperature stability of each endolysin differed was predictable and expected. In this study, although each endolysin was exposed to the indicated temperatures for one hour before measuring residual activity, it is anticipated that prolonged exposure would further decrease the residual activity. Accordingly, the observed higher efficacy of Lys10-24(13) in vivo suggests that temperature stability is one of the major factors in determining in vivo efficacy. Additional factors could include resistance to proteolytic cleavage, concentration of inorganic ions in the surrounding environment, and pH.

There are currently thousands of genes encoding putative endolysins found in GenBank. The majority do not demonstrate intrinsic antibacterial activities when supplied as recombinant proteins. As novel recombinant endolysins targeting Gram-negative pathogens are increasingly reported, more features in common are likely to be observed. As such, it is anticipated that amino acid-sequence based predictions of activity will become possible at some point in the future, and this current study takes a tentative first step towards the provision of data for the construction of associated databases.

## Figures and Tables

**Table 1:** Minimum inhibitory concentrations of the three endolysins against various bacterial strains. Shaded boxes indicate that the MIC was higher than the concentration range tested.

Strains	MIC (μg/mL)		
	CecA-Lys10-24(13)	CecA-LysPBEC30	CecA-LysPBEC56
*E. coli*			
ATCC 8739	8	16	16
K-12 MG1655	4	32	4
ATCC 25922	4	16	8
ATCC 700927	>64	>64	>64
F611	>64	>64	>64
F703	64	>64	>64
F716	16	32	32
F852	16	>64	32
F859	8	64	64
F862	4	4	16
F906	32	>64	64
*P. aeruginosa*			
PAO1	8	32	32
ATCC 15522	8	>64	16
ATCC 15692	8	32	16
ATCC 13388	4	4	8
ATCC 10145	>64	>64	>64
ATCC 9027	64	>64	64
ATCC 27853	16	>64	16
F147	16	32	32
F102	32	>64	>64
F265	>64	>64	>64
F388	16	16	16
F341	16	32	32
*A. baumannii*			
ATCC 19606	4	4	8
ATCC 17978	32	16	32
KACC 13090	8	32	16
KACC 14233	16	32	32
K3680	16	64	16
K643	16	64	64
F4	>64	32	64
F15	16	64	16
F65	64	64	32
F66	16	8	16
F67	8	16	16
F68	32	64	64
F69	64	64	64
*K. pneumoniae*			
KCTC 2208	4	4	4
KCTC 2296	8	8	16
KCTC 2246	16	>64	16
ATCC 700603	16	>64	>64
F120	32	16	64
F147	16	>64	64
F126	8	8	16
F85	32	>64	>64
*E. aerogenes*			
F276	16	>64	>64
*E. cloacae*			
ATCC 13047	8	8	32

## Data Availability

The original contributions presented in the study are included in the article. Further inquiries can be directed to the corresponding author.

## Supplementary Materials

The following supporting information can be downloaded at: https://www.mdpi.com/article/10.3390/v15030679/s1, Figure S1: Coding sequence of LysEC508M and analysis of its predicted helix structures. Note the absence of C-terminal amphipathic helix with cationic moiety.
