# Supplementary figures and images for: Characterization of Three Different Endolysins Effective against Gram-Negative Bacteria
Source: Viruses. 2023 Mar 4;15(3):679. doi: 10.3390/v15030679 (PMC10053066; doi:10.3390/v15030679)

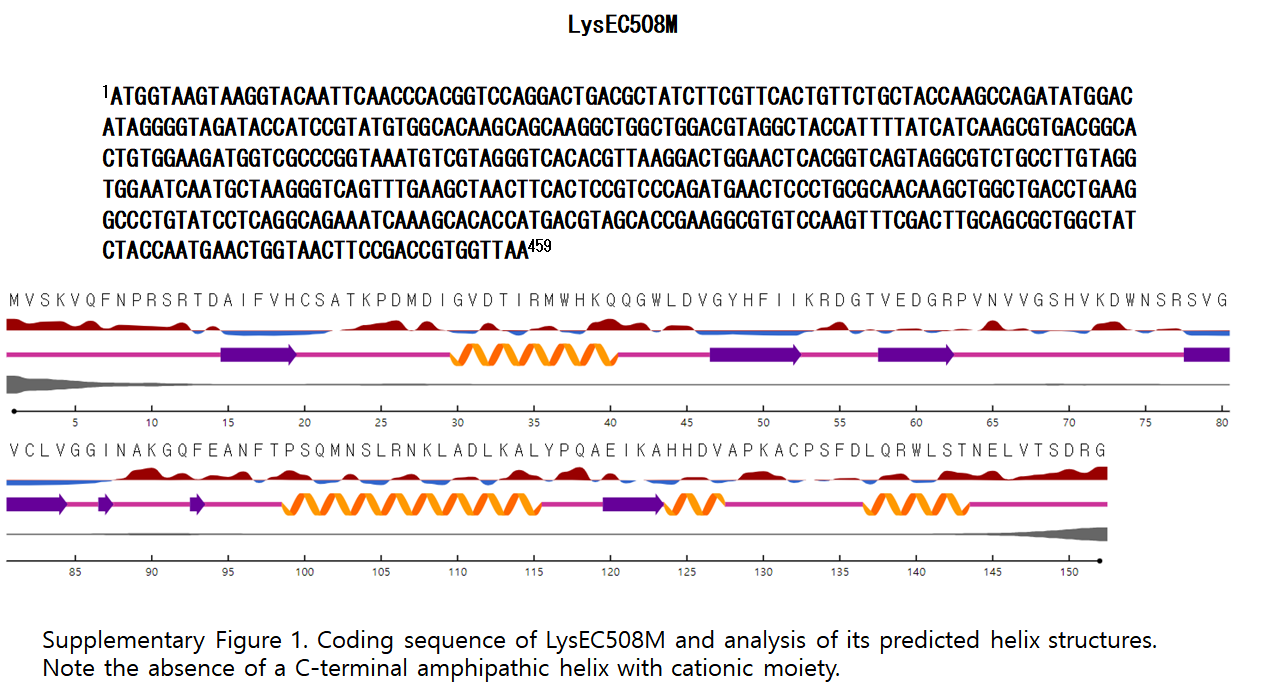

Supplement: Supplementary file 1 [file viruses-15-00679-s001.zip › viruses-2143419-supplementary.png]
